# Comprehensive cardiovascular assessment of children with chronic kidney disease using exercise cardiac magnetic resonance imaging

**DOI:** 10.1186/1532-429X-18-S1-P157

**Published:** 2016-01-27

**Authors:** Mun Cheang, Nathaniel J Barber, Jennifer A Steeden, Grzegorz T Kowalik, Kjell Tullus, Daljit Hothi, Vivek Muthurangu

**Affiliations:** 1 University College London, London, United Kingdom; 2 Department of Cardiovascular Imaging, Great Ormond Street Hospital, London, United Kingdom; 3 Renal Department, Great Ormond Street Hospital, London, United Kingdom

## Background

Children with chronic kidney disease (CKD) have a significantly increased cardiovascular (CV) risk. The reasons for this remain unclear. However, abnormalities of systolic and diastolic myocardial function, the vascular system and autonomic dysfunction have been implicated. Conventional investigations like resting echocardiography offer limited assessment of the CV system (CVS). Comprehensive evaluation of the CVS using advanced cardiac magnetic resonance imaging (CMR) is feasible. Furthermore, exercise can unmask autonomic dysfunction not evident at rest. Hence, this study will use CMR to characterize CV response to isometric exercise in pediatric CKD.

## Methods

10 children (age 13 ± 3 years) with CKD stages 2-4 and 10 matched healthy volunteers (HV) underwent an isometric stress CMR. This involved 3 minutes of static bicep flexion against a fixed weight (35% of maximum bicep strength). Left ventricular (LV) structure and function was assessed using real-time radial kt-SENSE sequence. Vascular haemodynamic properties of the aorta were evaluated with prospectively triggered segmented spiral phase contrast sequence and non-invasive blood pressure (BP) measurements. LV diastolic function was assessed by measuring cardiac timing intervals and LV inflow-outflow velocities using a high temporal resolution real-time spiral UNFOLDed-SENSE phase contrast sequence. Data was acquired at rest, exercise and recovery. In addition, a high temporal and spatial resolution, retrospectively gated, spiral tissue phase mapping sequence was used to assess radial and longitudinal LV function at rest. Image analysis was performed using in-house plug-ins for OsiriX software.

## Results

At rest, patients had a significantly (p < 0.04) higher mean arterial BP (MAP: CKD 89 ± 13, HV 75 ± 7 mmHg), systemic vascular resistance (SVR: CKD 20.5 ± 4.7, HV 14.4 ± 3.4 wood units) and lower total aortic compliance (TAC: CKD 1.25 ± 0.28, HV 1.61 ± 0.33 mls/mmHg). Myocardial longitudinal systolic velocity was lower in CKD (S': CKD 3.70 ± 0.70, HV 5.33 ± 1.81 cm/s). There was no difference in LV mass, ejection fraction (EF), cardiac output (CO), myocardial performance index (Tei), diastolic parameters (E/A ratio, isovolumic relaxation time & myocardial radial and longitudinal diastolic velocities) and myocardial radial systolic velocities. Isometric exercise provoked a modest increase in MAP (13 ± 11 mmHg), heart rate (HR 14 ± 18 beats per min), and CO (0.86 ± 1.49 ml/min). However, there were no differences in CV reactivity (heart rate & MAP) and LV function (EF & CO) between CKD and HV in exercise.

## Conclusions

Raised SVR and reduced TAC are present in early CKD and are the causes of hypertension in this population. This precedes diastolic dysfunction and LV hypertrophy. Reduction in longitudinal systolic velocity may be an early indicator of LV longitudinal function impairment. The CV and autonomic response to isometric stress is preserved in children with CKD despite these vascular abnormalities.

**Figure 1:**
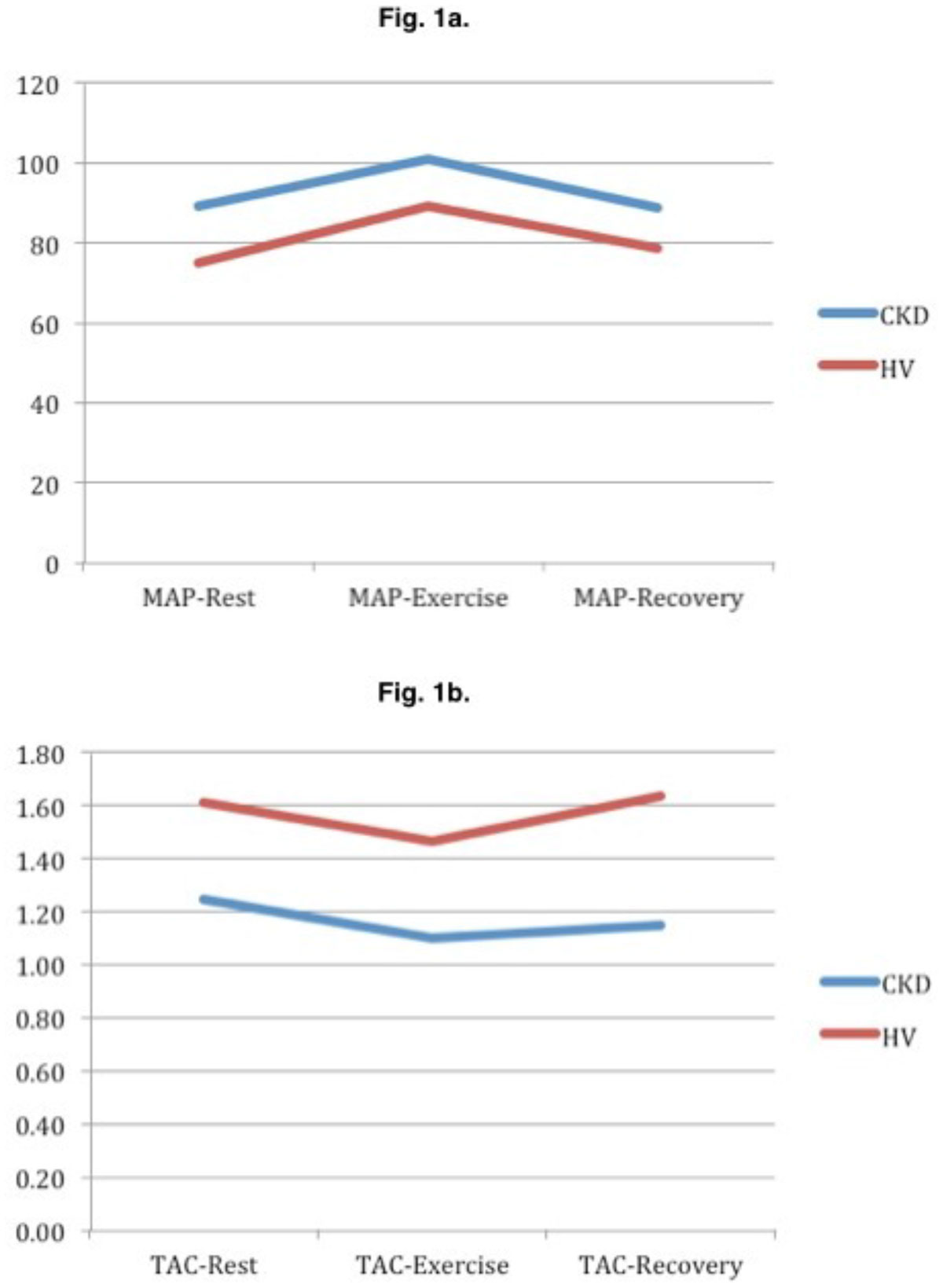
**Changes in mean arterial BP (Figure 1a) and compliance (Figure 1b)**.

